# Glycated Hemoglobin Electrochemical Immunosensor Based on Screen-Printed Electrode

**DOI:** 10.3390/bios12100902

**Published:** 2022-10-21

**Authors:** Yuliang Zhao, Hongyu Zhang, Yang Li, Xiaoai Wang, Liang Zhao, Jianghong Xu, Zhikun Zhan, Guanglie Zhang, Wen Jung Li

**Affiliations:** 1 School of Control Engineering, Northeastern University at Qinhuangdao, Qinhuangdao 066004, China; 2 Department of Mechanical Engineering, City University of Hong Kong, Hong Kong SAR 999077, China; 3 Key Laboratory of Intelligent Rehabilitation and Neuromodulation of Hebei Province, School of Electrical Engineering, Yanshan University, Qinhuangdao 066004, China; 4 Dalian Institute of Measurement and Testing, Dalian 116033, China; 5 Qinhuangdao Hospital of Traditional Chinese Medicine, Qinhuangdao 066004, China; 6 School of Computer and Communication Engineering, Northeastern University at Qinhuangdao, Qinhuangdao 066004, China; 7 City University of Hong Kong Shenzhen Research Institute, Shenzhen 518057, China

**Keywords:** glycated hemoglobin (HbA1c), screen-printed electrode (SPE), electrochemical immunosensor, differential pulse voltammetry (DPV), cyclic voltammetry (CV)

## Abstract

An electrochemical HbA1c sensor with high sensitivity and good specificity is proposed based on the electrochemical immune principle. The reproducibility and conductivity of the electrode are improved by depositing gold nanoparticles (AuNPs) on the surface of the screen-printed electrode (SPE). The HbA1c antibodies are immobilized on the surface of the modified electrode by adsorption to capture the HbA1c in the sample. The hindering effect of HbA1c on the electrode transfer reaction was exploited as the HbA1c detection mechanism. The electrode’s properties were characterized by electrochemical impedance spectroscopy (EIS), and the measurement properties of the electrode were analyzed using differential pulse voltammetry (DPV) and cyclic voltammetry (CV). The experimental results show that the peak current signal of the electrochemical immunosensor produced a linear response to HbA1c in the concentration range of 20–200 μg/mL, a linear relationship coefficient of 0.9812, a detection limit of 15.5 µg/mL, and a sensitivity of 0.0938 µA/µg·mL^−1^. The sensor delivered satisfactory repeatability, stability, and anti-interference performance. Due to its small size, high sensitivity, and wide linear detection range, it is expected to play a significant role in managing diabetes at home.

## 1. Introduction

Diabetes is a metabolic disease characterized by hyperglycemia, which is caused by defective insulin secretion or impaired function. It then induces various functions of the body, especially leading to the dysfunction of the eye, kidney, heart, blood vessels, and nerves [1,2]. It is estimated that the number of people with diabetes worldwide will increase from 422 million in 2016 to 550 million in 2030 [3]. Blood glucose, insulin, C-peptide, and glycated hemoglobin (HbA1c) are commonly used monitoring indicators for the diagnosis and management of diabetes [4,5,6]. However, the contents of blood glucose, insulin, and C-peptide usually fluctuate significantly in a short time, which is only suitable for short-term monitoring. HbA1c is a stable glycated protein formed by the reaction of glucose with hemoglobin (Hb) in red blood cells [7,8]. It can reflect the average blood glucose level in the past 2–3 months without interference from the external environment [9]. HbA1c-based diabetes diagnosis requires no fasting and remains effective for a more extended period, making it an effective long-term indicator for diabetes monitoring [10]. The physiological levels of HbA1c range from 3 to 13 µg/mL in human blood samples [11]. In 2010, the American Diabetes Association (ADA) recommended an HbA1c level of 6.5% or higher to diagnose diabetes [12]. This criterion can extensively screen abnormal HbA1c levels in a large number of high-risk groups, and thus can serve as an effective method for the large-scale early screening of diabetes [13]. 

Liquid chromatography [14], electrophoresis [15], affinity chromatography [16], ion-exchange chromatography [17], and immunoassay [18] are among the most used methods to determine the level of HbA1c in clinical laboratories. However, the efficiency of these methods relies on professional equipment and personnel, and the detection process is quite complex [19]. In contrast, electrochemical methods are easy to operate and require no professional equipment, and the costs are lower [20,21]. Existing studies have shown that HbA1c level measurement based on the electrochemical principle has the advantages of linear output, low power consumption, and reusability. The selectivity of HbA1c only depends on the performance of the sensor electrode. Most electrochemical methods can achieve fast, micro-scale, and low-cost measurement, exhibiting superiority over other testing methods in terms of point-of-care testing (POCT) [22].

As currently reported, the most common detection techniques and signals for HbA1c in electrochemistry are amperometry, voltammetry [23], potential [24], and impedance [25]. These three types of sensors include sensing interface modifications and HbA1c capture. Interface-modified substances include metal oxides [26], graphene [27], nanomaterials [28], etc.; captured materials include boric acid and their derivatives [29,30], ferrocene derivatives [31], DNA aptamers [32], antibodies [33], and so on. Zhou et al. [34] reported a voltammetric sensor with composites of phenylboronic acid-modified pyrroloquinoline (PBA-PQQ) and reduced graphene oxide (RGO) for determination of HbA1c, and it required an additional step of blood sample pretreatment to remove glucose and other glycated proteins. Surachet et al. [35] selected and purified DNA aptamers to determine HbA1c content by Systematic Evolution of Ligands by EXponential (SELEX); however, the purification of aptamers is complex. HbA1c sensors based on antibodies have the characteristics of good specificity and reliability, it have attracted the research community’s attention. Xue et al. [36] introduced gold nanoparticles based on the self-assembled monolayer (SAM) and incubated anti-HbA1c on the surface of the test strip, and the potential signal of the sensing integrated chip showed a logarithmic response to 4–24 µg/mL of HbA1c. In order to make the electrochemical immunosensor design easier, and reduce operation steps, Wang et al. [37] incubated glycated hemoglobin antibody (anti-HbA1c) on the SPE to capture HbA1c and catalyzed the reduction reaction of H_2_O_2_ to convert chemical signals into current signals, which was based on the peroxidase activity of HbA1c. Alireza et al. [38] employed a self-assembled monolayer of 3-mercaptopropionic acid (MPA) to covalently immobilize anti-HbA1c on the surface of gold electrodes. In addition, using undiluted human serum as the test medium, the biosensor presented an excellent linear behavior in the range of 100–250 mg/mL of HbA1c and broadened the scope of detection of HbA1c.

Committed to the preparation method, fast and simple response results, and wide detection range, based on the principle of electrochemical immunity, we propose a low-cost screen-printed three-electrode system for the high-sensitivity detection of HbA1c by gold nanoparticles modification, immune coupling, and electrochemical measurement (as shown in Table 1). The sensor can obtain a strong response signal in a low-concentration HbA1c solution with high specificity, requiring no labeling, and with a simple procedure. It has a small size, low cost (CNY 50), wider linear detection range, and low sample volume (10 µL). If integrated with portable electrochemical detection devices, the screen-printed electrode can potentially evolve into an effective and economic diabetes management and monitoring device for home users.

## 2. Materials and Methods

### 2.1. Material and Instrumentation

The human HbA1c antibody (anti-HbA1c) and human glycated hemoglobin (HbA1c) were purchased from Fitzgerald (United States). The chloroauric acid (HAuCl_4_), potassium sulfate (K_2_SO_4_), Tween–20 (Tween–20), and potassium ferricyanide (K_3_[Fe(CN)_6_]) used in the pretreatment, modification, and measurement of the electrode were purchased from Sinopharm (Sinopharm, Beijing, China). Sulfuric acid (H_2_SO_4_), phosphate buffer (PBS, 0.01 M, PH 7.4), hydrogen peroxide (H_2_O_2_), and other chemicals were all analytical grade and used without further purification. Antibody, antigen, and Tween-20 solution were prepared with PBS buffer, and other solutions were prepared with ultra-pure water.

**Figure 1:**
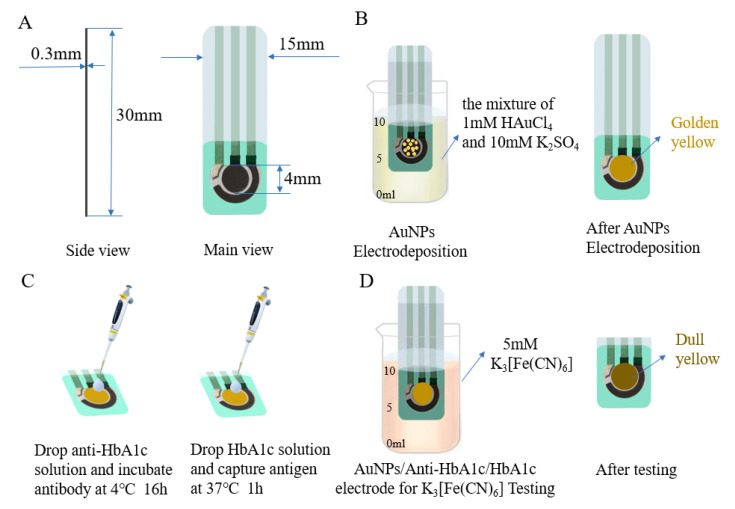
(**A**) Dimensions of the SPE. (**B**) Electrodeposition of SPE in the mixture of 1 mM HAuCl_4_ and 10 mM K_2_SO_4_. (**C**) Anti-HbA1c incubation and capture HbA1c. (**D**) AuNPs/anti-HbA1c/HbA1c electrode for K_3_ [Fe(CN)_6_] testing.

All electrochemical experiments were carried out in the CHI660e Electrochemical Workstation (Shanghai Chenhua Co., Shanghai, China). The SPE as an electrochemical sensing component was purchased from Qingdao Glassy Carbon Technology Co., Ltd. The base of the working electrode and counter electrode were carbon electrodes, and the base of the reference electrode was a silver electrode. As shown in Figure 1A, the electrode was 30 mm long, 15 mm wide, and 0.3 mm thick; and the diameter of the working electrode was 4 mm. All experiments, consisting of DPV, CV, and EIS, were carried out in a 10 mL beaker.

### 2.2. HbA1c Electrochemical Immunoassay Scheme

**Figure 2:**
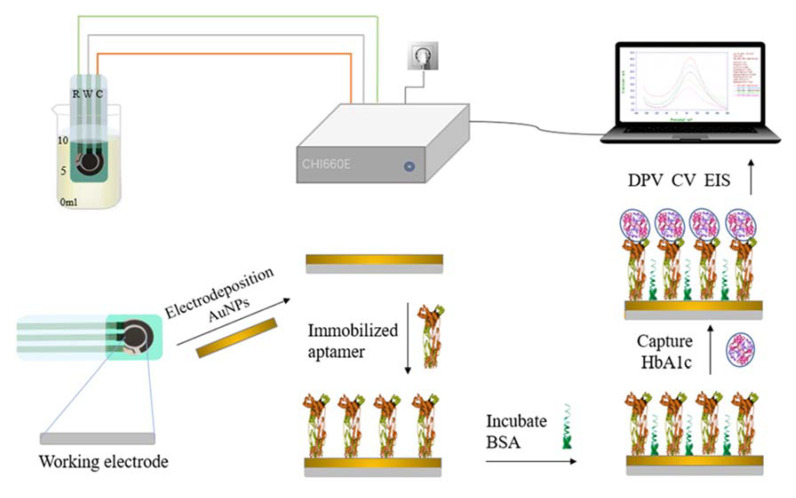
Schematic diagram of the proposed electrochemical HbA1c immunosensor.

In this paper, electrochemical immunoassay was used for the quantitative detection of HbA1c. The screen-printed electrode (SPE) modification and measurement process is shown in Figure 2. The process involved four steps: electrode pretreatment and modification, antibody immobilization, antigen capture, and electrochemical measurement and analysis. Gold nanoparticles were modified on the working electrode by electrochemical deposition to improve the area and reproducibility of the electrode [39]. The characteristics of the electrode were characterized by electrochemical impedance spectroscopy (EIS). Anti-glycated hemoglobin was deposited on the electrode surface by electrostatic adsorption. The HbA1c was captured based on the specific coupling characteristics of the antibody and antigen [40]. Eventually, HbA1c was quantitatively measured by differential pulse voltammetry (DPV) and cyclic voltammetry (CV). 

#### 2.2.1. Pretreatment and Modification of the SPE

Pretreatment of an electrode can significantly improve the volt-ampere characteristics of the electrode and enhance its activity [41]. The SPE was pretreated using the following procedure: first, the SPE was immersed in a 0.5M H_2_SO_4_ solution; then, it was scanned by CV at a voltage ranging from –0.6 to 1.0 V until a stable signal was obtained; finally, the surface of the scanned electrode was washed with ultra-pure water to remove the residual substances in the pretreatment solution and minimize its influence on the subsequent measurement process. 

The electrode was modified by CV, and the pretreated electrode was immersed in the electrolyte formed by the mixture of 0.001 M HAuCl_4_ and 0.01 M K_2_SO_4_ for electrochemical measurement. When the SPE was submerged in the electrodeposition solution, the surface of the working electrode would turn golden yellow (as shown in Figure 1B). The potential window range of the external electric field was set to –0.5–1.1 V, the sweep segments to 76, and the scanning rate to 100 mV/s by default. After the scanning, the electrode was cleaned with ultra-pure water and dried. In the modification process, the performance of the electrode could be adjusted by changing the CV deposition conditions and the concentration of the chloroauric acid solution. Finally, the electrode was immersed in the commonly used electrolyte potassium ferricyanide (with a concentration of 5 mM), and the degree of modification was tested by CV and DPV. 

#### 2.2.2. Immobilization of Antibodies on the Electrode and Specific Binding of Antigens

A layer of gold film with nanoscale spotty distribution was attached to the surface of the modified SPE, which greatly increased the adhesion area of the antibody, thereby increasing the antibody coating amount and improving the sensing performance of the electrode [42].

The antibodies were immobilized on the electrode by a simple adsorption process. An AuNPs-modified SPE was taken to the surface of this working electrode, and 10 μL of 500 μg/mL HbA1c antibody solution was dropped. Then, the electrode was incubated at 4 ℃ for 16 h. After the incubation, the electrode surface was washed with PBS buffer to remove the unbound antibodies. Then, 50 μL of bovine serum albumin (BSA) was applied to the three-electrode region and sealed at 37 ℃ for 2 h to seal the blank sites on the electrode surface that did not bind to the antibody. In addition, the electrode was again rinsed with PBS buffer and dried naturally at room temperature. At this point, the immune electrode was obtained.

A series of antigen solutions of different concentrations were prepared, and 10 μL of each was applied to the surface of the working electrode and incubated at 37 ℃ for 1 h (as shown in Figure 1C). Then, the electrode was washed with PBS buffer containing 0.1% Tween–20 5 times and thoroughly washed with PBS buffer to remove antigens that did not bind to antibodies on the electrode surface. Finally, the AuNPs/anti-HbA1c/HbA1c-electrode was immersed in 5mM K_3_ [Fe(CN)_6_] for electrochemical measurement (as shown in Figure 1D). To ensure that enough antigens in the sample to be tested are successfully captured by the antibodies immobilized on the electrode surface, the number of antibody molecules should be larger than the number of antigen molecules. The molar concentration of the anti-HbA1c solution used in this paper was 1.13–22.6 times that of the HbA1c solution. In addition, since the half-life of glycated hemoglobin is 36 days, the modification time will not lead to glycated hemoglobin decomposition or other changes [43].

#### 2.2.3. Parameters Setup for Electrochemical Measurement

When the immune electrode fully captures the antigen molecules in the sample, the immune electrode can produce a specific electrical response. A small amount of antigen information can be detected through the quantitative analysis of this phenomenon by electrochemical measurement. DPV, CV, and EIS were used to comprehensively analyze the characteristics of the modified electrode, the immune electrode, and the electrode bound with antigens to obtain the characteristics of each type of electrode and quantitatively detect the content of HbA1c in the sample. HbA1c can catalytically reduce both H_2_O_2_ [44] and K_3_[Fe(CN)_6_] [45] and generate current signals. Different concentrations of HbA1c yield different electrochemical responses. The DPV test was carried out in K_3_[Fe(CN)_6_], and the CV test was carried out in H_2_O_2_.

The changes in the surface characteristics of each type of electrode were analyzed comprehensively by CV and EIS. In CV detection, the potential range was from –0.7 to 1.0 V, the scanning rate was 100 mV/s, and the sampling interval was 1 mV. For the EIS parameters, the frequency range was set to 0.01 to 105 Hz and the amplitude to 5 mV. The DPV and CV methods were used to analyze the HbA1c in the samples quantitatively. For DPV parameters, we set the potential range as –0.2 to 0.3 V, the amplitude to 50 mV, and the step potential to 4 mV.

## 3. Results

### 3.1. Electrode Performance Evaluation and Analysis

**Figure 3:**
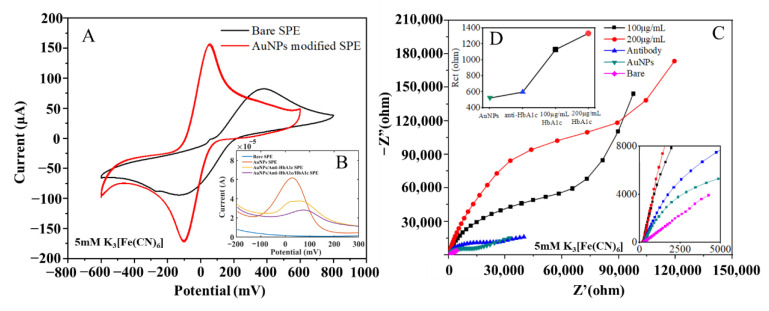
(**A**) CV response of SPEs before and after AuNPs modification. (**B**) DPV response of bare SPE, AuNPs SPE, AuNPs/anti-HbA1c SPE and AuNPs/anti-HbA1c/HbA1c SPE. (**C**) Complex impedance characteristics of electrodes in different states. (**D**) Charge-transfer resistance of electrodes in different states.

To quantitatively evaluate the performance changes of the electrode before and after modification, a CV test scheme was adopted, with a 5 mM K_3_ [Fe(CN)_6_] solution used as the electrolyte. The redox peak current increased, which indicated that the conductivity of the electrode was improved. The CV curves of the unmodified electrode and the AuNPs-modified electrode are shown in Figure 3A. It can be seen that the difference between the upper and lower peak potentials of the bare electrode was 500 mV, and its peak current amplitude was only 80 μA, which meant its conductivity was poor and inadequate for subsequent measurement experiments. After modification by the electrodeposition method with optimized experimental conditions, the peak potential difference of the electrode decreased to 120 mV, and its peak current increased to 160 μA, indicating that its conductivity improved by 50%. Due to the hindering effect of HbA1c on the ferricyanide/ferricyanide electron transfer reaction, the anti-HbA1c on the surface of the AuNPs leads to a gradual weakening of the signal generated by the K_3_[Fe(CN)_6_]/K_4_[Fe(CN)_6_] redox couple reaction [38].

**Figure 4:**
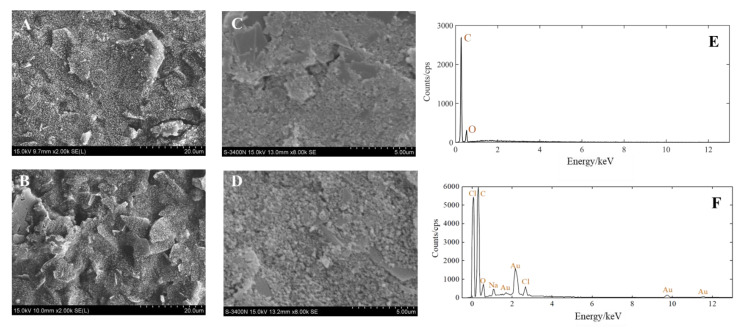
(**A**) SEM image of the SPE (2000 times). (**B**) SEM image of the SPE after AuNPs modification (2000 times). (**C**) SEM image of the SPE (8000 times). (**D**) SEM image of the SPE after AuNPs modification (8000 times). (**E**) EDX spectrum of the bare electrode. (**F**) EDX spectrum of the AuNPs-modified electrode.

The morphology and element composition of the AuNPs-SPE were investigated utilizing scanning electron microscopy (SEM) and energy dispersive X-ray spectroscopy (EDX). The SEM images of the SPE before and after deposition and the EDX images of the AuNPs-SPE are shown in Figure 4. At 2000 times magnification, the surface of the bare electrode was flat (Figure 4A). In contrast, after deposition, the surface of the modified electrode had different flakes of particles and large bulges (Figure 4B). At 8000 times magnification, there were more spherical substances on the surface of the modified electrode (Figure 4D) than on the bare electrode (Figure 4C). This indicates that the target material (AuNPs) was attached to the bare electrode. Furthermore, the elements present on the bare-SPE and AuNPs-SPE surface were analyzed using EDX. The working electrode of bare SPE was a carbon electrode, as shown in Figure 4E. The working electrode of AuNPs-SPE is shown in Figure 4F. There were O, Na, and Cl elements on the AuNPs-SPE because PBS was used to rinse the electrode and HAuCl_4_ solution was used in the electrodeposition of the electrode, which did not affect the subsequent tests. Au was present on the surface of the working electrode. Therefore, the above results demonstrated that the SPE was successfully modified by gold nanomaterials.

Electrochemical impedance spectroscopy (EIS) is a powerful, non-destructive, and informative technology that can provide the impedance characteristics of an electrode surface [46]. The impedance spectrum test results for electrodes in each stage are shown in Figure 3B, with the frequency range of 1 × 10^−2^ to 1 × 10^6^ Hz, and the tests were carried out in 5 mM K_3_[Fe(CN)_6_]. In the order from the smallest radius of the characteristic impedance semicircle to the largest, the curves represent the complex impedance characteristics of the bare electrode, the modified electrode, the immune electrode, the electrode bound with 100 μg/mL antigens, and that with 200 μg/mL antigens. 

In Figure 3C, the semicircle corresponds to the high-frequency region, showing the charge control process [47]; the diameter of the semicircle represents the electron-transfer resistance on the electrode surface; and the straight line corresponds to the low-frequency region, representing the diffusion control process. From the semicircle of the characteristic impedance, it can be seen that the bare electrode was unmodified, and the electron transfer impedance was large, so there remained only the diffusion process. For the modified electrode, due to an additional layer of gold nanoparticles on the surface, the surface area was greatly increased, thus improving the interaction between the electrode and the redox pair, improving the electron transfer ability, and minimizing the electron transfer impedance. Therefore, the radius of the characteristic impedance semicircle was the smallest. After the antibodies were immobilized on the surface of the gold electrode, the electron transfer process was hindered, which led to increased electron transfer impedance on the surface of the gold electrode. With the binding of the antigens and antibodies, the electron transfer was further hindered, and the equivalent impedance of the electrode surface became larger than that of the immune electrode, the higher the antigen concentration, the larger the radius of the characteristic impedance semicircle. In the equivalent circuit, the constant phase element was used to replace the double-layer capacitance. In Figure 3C and Figure 4D the semicircle radius of the Nyquist curve gradually increases, and the charge transfer resistance (R_ct_) gradually increases from the modified electrode to the immune electrode and antigen electrode. 

### 3.2. Electrochemical Measurement of the Concentration of HbA1c Solution

The concentration of HbA1c was quantitatively analyzed by DPV and CV, respectively. HbA1c standard solution was prepared in two concentration series for measurements by DPV and CV methods, respectively. Specifically, antigen solutions with concentrations of 10 μg/mL, 20 μg/mL, 60 μg/mL, 80 μg/mL, 100 μg/mL, 150 μg/mL, and 200μg/mL were used to establish the standard curve and carry out the measurement in the 5 mM K_3_[Fe(CN)_6_] by DPV. Double gradient antigen solutions with concentrations of 12.5 μg/mL, 25 μg/mL, 50 μg/mL, 10 μg/mL, and 200 μg/mL were used to draw the standard curve in 1 mM H_2_O_2_ by CV. The concentration of the antibody solution was 500 μg/mL in the measurement process.

**Figure 5:**
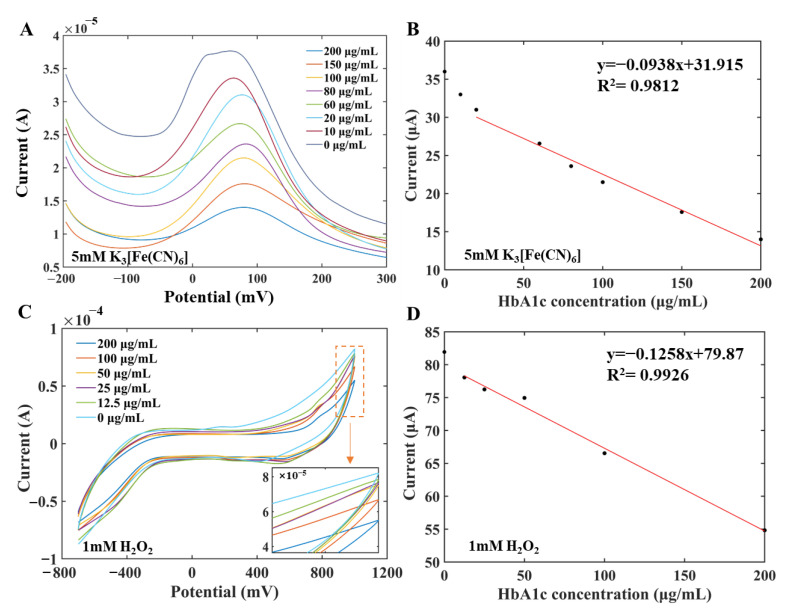
Measurement curves and linear fitting diagram of the modified working electrode for different concentrations of HbA1c. (**A**) DPV measurement curves of the modified working electrode for different concentrations of HbA1c. (**B**) Linear fitting diagram of DPV peak current curves. (**C**) CV measurement curves of the modified working electrode for different concentrations of HbA1c. (**D**) Linear fitting diagram of CV current curves.

Figure 5A,B show the current–potential curve and the established standard curve measured by DPV, respectively. It can be seen from Figure 5A that as the concentration of HbA1c in the sample increased, the number of antibodies bound to antigens on the electrode surface increased, but the electron transfer ability decreased, which led to a smaller response current. When the concentration of HbA1c was in the range of 20–200 μg/mL, there was a linear relationship between the peak current (μA) and the concentration of HbA1c (μg/mL) on the current-potential curve of DPV. The fitting equation was y = –0.0938x + 31.915, R^2^ = 0.9812, the detection limit was 15.5 μg/mL, and the sensitivity was 0.0938 µA/µg·mL^−1^. The limit of detection (LOD) was calculated according to Equation (1):

LOD = k·s/b(1)

where k = 3.3, s is the standard deviation of 9 to 12 measurements in blank samples, and b is the slope of the fitted curve, which is also the sensitivity [48].

Figure 5C,D show the CV curve and the standard curve measured by CV, respectively. As shown in Figure 5C, as the concentration of HbA1c in the sample increased, the number of antibodies bound to antigens on the electrode surface increased, but the electron transfer ability of the electrode decreased, which led to a smaller response current. The current values at 1000 mV of different concentration CV curves were selected to characterize the HbA1c concentration information. There was a linear relationship between the oxidation current I (μA) and the concentration of HbA1c solution c (μg/mL). The fitting equation was I = –0.1258c + 79.87, R_2_ = 0.9926, and the sensitivity was 0.1258 µA/µM. The experimental results demonstrated that the measurements obtained by CV analysis were basically consistent with those obtained by DPV and can be used for quantitative analysis.

### 3.3. Evaluation of HbA1c Measurement Performance

**Figure 6:**
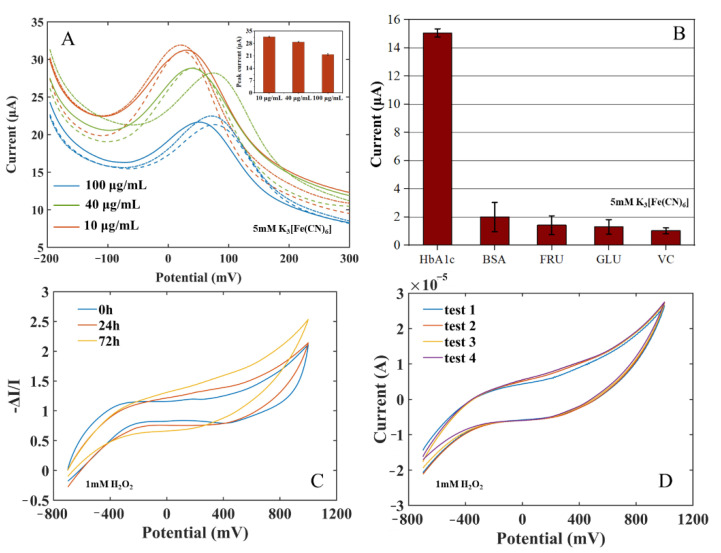
(**A**) Repeatability measurement: current of the immune electrodes to HbA1c of 10 μg/mL, 40 μg/mL, 100 μg/mL, and 150 μg/mL (insert: peak current comparison). (**B**) Specificity measurement: current of the immune electrodes to HbA1c, BSA, GLU, FRU, and VC. (**C**) Long-term measurement: 0 h, 24 h, 72 h. (**D**) Repeated experiments at 72 h.

To evaluate the repeatability, accuracy, and stability of the immune electrode in HbA1c measurement, three parallel tests were carried out on HbA1c solutions with concentrations of 10 μg/mL, 40 μg/mL, and 100 μg/mL using an HbA1c biosensor (as shown in Figure 6A). The mean value of the three measurements was taken as the final measurement result, and the Relative Standard Deviation (RSD) (RSD = standard deviation/current mean) of the sensor response current were calculated. The current RSD represents the discrete degree of the three measurements of the sensor to indicate the repeatability of measurement by the sensor. The results indicated that the proposed electrochemical HbA1c sensor has good repeatability and accuracy. The stability of the electrode was also measured for 72 h. The long-term response data are shown in Figure 6C and the intra-batch response data are shown in Figure 6D. When the electrode was stored at 4 ℃ for 24 h, the response current varied by about 1.03%. After 72 h, the response current varied by about 10.47%. Repetitive experiments at 72 h showed that the intra-batch coefficient of variation was 1.61%. The results show that the anti-HbA1c SPE still had good stability after long-term storage. 

In order to verify the specificity of the immune electrode and the characteristics of anti-false-positive interference, four substances with high protein and sugar properties in blood were selected to carry out three parallel experiments. The solutions of the four substances and their concentrations are as follows: bovine serum albumin (BSA, 1 mg/mL), fructose (FRU, 0.1 mol/L), glucose (GLU, 0.1 mol/L), and vitamin C (VC, 0.1 mol/L). The immune electrodes were used for immune incubation in the four solutions, and the DPV test was carried out in the potassium ferricyanide solution. The change in the peak current of each substance is shown in Figure 6B. The response current changes of the four interferents were much smaller than that of 150 μg/mL HbA1c, indicating that the immune electrode has good selectivity to HbA1c and strong anti-interference ability.

## 4. Conclusions

In this paper, an electrochemical immune sensor based on a gold nanoparticle-modified screen-printed electrode (SPE) was constructed for the quantitative measurement of HbA1c. The DPV results showed that the sensor had an excellent linear response in HbA1c solutions of concentrations ranging from 20 to 200 μg/mL, a detection limit of 15.5 µg/mL, and a sensitivity of 0.0938 µA/µg·mL^−1^. The CV results showed that the sensor had a good linear response in HbA1c solutions of concentrations ranging from 12.5 to 200 μg/mL and a sensitivity of 0.1258 µA/µg·mL^−1^. The sensor has the advantages of low cost, small sample volume (10 µL), small volume, no marking, good stability, and high detection accuracy. To sum up, this new HbA1c measurement platform is expected to develop into an effective diabetes management and monitoring device readily accessible to home users, playing a significant role in the early diagnosis of diabetes. 

## Figures and Tables

**Table 1:** Analytical Parameters for the Detection of HbA1c by Several Methods.

Electrode Materials	Method	Sensitivity	Detection Range	LOD	Interference	Reference
GCa/RGOb/PBA-PQQc	DPV	0.1255 µA/µg·mL^−1^	9.4–65.8 µg/mL	1.25 µg/mL	Hb, glucose, fructose, galactose	[34]
Array SPCE/AuNPs, thiol-modified aptamer	SWV	—	0.1–1000 ng/mL	0.2 ng/mL	—	[35]
SPE/anti-HbA1c/HbA1c	CV	0.199 µA/µg·mL^−1^	1–50 µg/mL	0.88 µg/mL	BSA, PSA, glucose	[37]
Test strip/AuNPs/dithiolate/anti-HbA1c	Potential	90.6 mV/log(C(HbA1c))	4–24 µg/mL	—	—	[36]
Gold electrode/MPA/anti-HbA1c/HbA1c	DPV	0.137 µA/µg·mL^−1^	7.5–20 µg/mL (PBS) 100–250 µg/mL (serum)	7.5 µg/mL	—	[38]
SPE/AuNPs/anti-HbA1c/HbA1c	DPV/CV	0.0938 µA/µg·mL^−1^ 0.1258 µA/µg·mL^−1^	20–200 µg/mL 12.5–200 µg/mL	15.5 µg/mL —	BSA, glucose, fructose, VC/—	This work

## Data Availability

Not applicable.
